# Building a ‘RAMP’ to Aging Research: Developing a Two-Year Summer High School Research Training Program

**DOI:** 10.1093/geroni/igaf122.3263

**Published:** 2025-12-31

**Authors:** Morgan Fique, Elizabeth Dennis, Anna Pudder, Linda Horn, Cara Felter, Bret Hassel

**Affiliations:** University of Maryland School of Medicine, Baltimore, Maryland, United States; University of Maryland School of Medicine, Baltimore, Maryland, United States; University of Maryland School of Medicine, Baltimore, Maryland, United States; University of Maryland, Baltimore County, Baltimore, Maryland, United States; University of Maryland, Baltimore County, Baltimore, Maryland, United States; University of Maryland School of Medicine, Baltimore, Maryland, United States

## Abstract

Participation in structured research and mentorship programs increases students’ likelihood of enrolling in college and graduate school while boosting their confidence in becoming researchers or clinicians. There is also a need to expand the aging research workforce; however, students are unlikely to be exposed to aging research at early educational levels. The Research and Mentoring Program (RAMP) is a summer research training program targeting 11th and 12th grade high school (HS) students from Baltimore City. This two-year program was designed to provide foundational knowledge across the translational research continuum while equipping students with basic lab techniques and clinical measurement skills (Year 1) to prepare them for a mentored summer research experience (Year 2) with faculty conducting aging-focused research from the University of Maryland Claude D. Pepper Older Americans Independence Center, University of Maryland Greenebaum Comprehensive Cancer Center (UMGCCC) and Baltimore Geriatric Research, Education, and Clinical Center (GRECC) within the VA Maryland Health Care System. In turn, RAMP faculty and near peer mentors receive mentoring training to enhance their experience in working with HS trainees. Scholars are compensated both summers through a partnership with Youthworks, a workforce development program funded by Baltimore City. This study tests the feasibility of developing and implementing a scalable training and education program that can be disseminated to other academic institutions providing a path to aging-focused research and clinical career pathways for HS students.

